# The Association of Adiponectin Gene Promoter Variations with Non-Small Cell Lung Cancer in a Han Chinese Population

**DOI:** 10.1371/journal.pone.0127751

**Published:** 2015-05-27

**Authors:** Yingfu Li, Yueting Yao, Xu Qian, Li Shi, Jingxian Zhou, Qianli Ma, Yufeng Yao

**Affiliations:** 1 Department of Geriatrics, The No.1 Affiliated Hospital of Kunming Medical University, Kunming, 650032, China; 2 Institute of Medical Biology, Chinese Academy of Medical Sciences & Peking Union Medical College, Yunnan Key Laboratory of Vaccine Research & Development on Severe Infectious Disease, Kunming, 650118, China; 3 Department of Cardiothoracic Surgery, Yan`an Hospital of Kunming, Kunming, 650051, China; 4 Department of Thoracic Surgery, The No.3 Affiliated Hospital of Kunming Medical University, Kunming, 650118, China; H. Lee Moffitt Cancer Center & Research Institute, UNITED STATES

## Abstract

Recently, in vitro studies have demonstrated that adiponectin has antiangiogenic and tumor growth-limiting properties. Additionally, serum adiponectin levels have been associated with the risk of several cancers; specifically, serum adiponectin was significantly lower in lung cancer patients with advanced-stage disease. In this study, we examined the association of adiponectin gene promoter variations associated with adiponectin gene expression and plasma levels in non-small cell lung cancer (NSCLC) in a Han Chinese population. A total of 319 patients with NSCLC and 489 healthy individuals were recruited to evaluate the association of four adiponectin gene promoter single-nucleotide polymorphisms (SNPs) (SNP-12140G>A, SNP-11426A>G, SNP-11391G>A and SNP-11377C>G) with NSCLS risk. Additionally, we constructed haplotypes of these four SNPs and evaluated the association of these haplotypes with NSCLS risk. Our results showed that among these four SNPs, only SNP-12140G>A was associated with NSCLC risk(P<0.05). The haplotype analysis showed that no haplotype was associated with NSCLC after performing a Bonferroni correction (P>0.05). Additionally, an association analysis of the four SNPs stratified into pathologic stages I+II and III+IV showed that these SNPs did not exhibit significant differences between pathologic stages I+II and III+IV. Moreover, we did not observe any differences in allele and genotype frequency for these SNPs between adenocarcinoma and squamous cell carcinoma. Our results indicated that the G allele of SNP-12140may be a risk factor for NSCLC (OR = 1.516; 95% CI: 1.098–2.094) in this Han Chinese population.

## Introduction

Lung cancer is one of the leading causes of cancer deaths worldwide and has a 5-year survival rate of approximately 15%[1], and non-small cell lung cancer (NSCLC) accounts for approximately 80% of lung cancer cases [2]. In China, the incidence and mortality of lung cancer are estimated to be 0.7 and 0.6 million cases, respectively.

Adiponectin is an adipose tissue-secreted protein that acts as an endogenous insulin sensitizer by binding to insulin receptors[3]. Previous studies have shown that adiponectin is associated with obesity[4, 5], insulin resistance[5, 6] and type 2 diabetes[7, 8]. Recently, several studies have reported that adiponectin has antiproliferative and proapoptotic effects in breast cancer cell lines in vitro[9, 10]. Additionally, many studies have reported that lower adiponectin levels are associated with an increased risk of endometrial cancer[11–14], renal cancer[15], colon cancer[16] and breast cancer[10, 17–19]. The above results suggest that adiponectin may play an important role in tumor development and growth. In 2007, Petridou *et al*. found that serum adiponectin was not significantly different in patients with lung cancer compared with controls, though it was significantly lower in patients with advanced-stage disease, suggesting that adiponectin could be a potential marker for lung cancer progression[3]. Recently, Nigro *et al*. provided evidence for a direct effect of adiponectin on the proliferation and inflammation status of A549 cells, which supported the hypothesis that adiponectin plays a protective role in the lung and suggested that adiponectin could be a promising therapeutic target in lung diseases[20].

In 2006, Heid *et al*. reported that *adiponectin* promoter single-nucleotide polymorphisms (SNPs) are associated with adiponectin concentrations[21]. Laumen *et al*. later demonstrated that *adiponectin* promoter SNPs regulate *adiponectin* promoter activity[22]. Therefore, *adiponectin* promoter variations may have an impact on cancer risk.

We previously reported the association of two adiponectin gene promoter SNPs (SNP-12410G>A and SNP-11377C>G) with NSCLC risk in a Han Chinese population, and the results showed that SNP-12140G>A (rs266730) is associated with increased NSCLC risk[23]. To confirm these results, in this study, we increased the sample size (from 179 to 319 cases, from 242 to 489 controls) and added two other adiponectin gene promoter SNPs (SNP-11426A>G and SNP-11391G>A) to evaluate the association of these four adiponectin gene promoter SNPs, namely,SNP-12140G>A (rs266730), SNP-11426A>G (rs16861194), SNP-11391G>A (rs17200539) and SNP-11377C>G (rs266729), and their haplotypes with NSCLC risk in a Han Chinese population.

## Materials and Methods

### 1.1 Ethics statement

This protocol was conducted in accordance with the Declaration of Helsinki and was approved by the Institutional Review Boards of the No.1 and No.3 Affiliated Hospitals of Kunming Medical University. All participants provided written informed consent.

### 1.2 Subjects

The case group included 319 patients (213 males and 106 females) who were diagnosed with NSCLC at the No.1 and No.3 Affiliated Hospitals of Kunming Medical University from July 2012 to May 2014. The histological type of lung cancer was identified according to the World Health Organization (WHO 2004) classifications. The pathologic stage was determined according to the International System for Staging Lung Cancer [24]. Based on the pathomorphological reports, the NSCLC cases included adenocarcinoma (AC), squamous cell carcinoma (SCC), and adenocarcinoma and squamous cell carcinoma (AC+SCC). NSCLC patients with a prior history of primary cancer other than lung cancer were excluded from the current study. Additionally, individuals with hypertension, coronary heart disease and diabetes were also excluded from this study to avoid any potential interference from overlapping genes. Clinical characteristics and data, such as sex, age, family history of cancer, and histological type of cancer, were obtained. The healthy control group included 489 subjects (313 males and 176 females) who had no family history of NSCLC and were recruited from a population undergoing routine health checkups at the No.1 and No.3 Affiliated Hospitals of Kunming Medical University. All participants (NSCLC patients and healthy controls) self-reported as ethnic Hans and lived roughly within the same geographic region (Yunnan Province, China).

### 1.3 NP genotyping using TaqMan assay method

Genomic DNA was extracted from peripheral lymphocytes using a standard hydroxybenzene-chloroform method. Four *adiponectin* promoter SNPs, namely SNP-12140G>A (rs266730), SNP-11426A>G (rs16861194), SNP-11391G>A (rs17200539) and SNP-11377C>G (rs266729), were genotyped using PCR amplification with a TaqMan assay. Primers and probes were purchased from Applied Biosystems (Foster City, CA, USA). Selected PCR products were characterized by direct sequencing on a 3100 Genetic Analyzer (Applied Biosystems, Tokyo, Japan).

### 1.4 Statistical analysis

The allele and genotype frequencies of the four SNPs were calculated by the direct-counting method. Hardy-Weinberg equilibrium (HWE) was tested for the SNPs in both the NSCLC and control groups. The linkage disequilibrium (LD) and haplotype frequencies (deduced from the phenotype) were calculated based on the genotyping results by the expectation-maximization algorithm in SHEsis software[25, 26].A χ^2^ test was used to determine differences in allele, genotype and haplotype frequencies between the NSCLC and control groups, and the odds ratios (OR) with associated 95% confidence intervals (CIs) of allele-specific risks were calculated. Bonferroni correction was used for testing multiple comparisons. The association between each SNP and NSCLC was analyzed for the mode of inheritance using SNPStats software [27]. The Akaike information criterion (AIC) and Bayesian information criterion (BIC) were used to determine the best-fit model for each SNP. The statistical power was calculated using PS Software[28]. The statistical analyses were performed using SPSS 13 (Chicago, IL). A P value less than 0.05 was considered statistically significant.

## Results

### 1.1 Subject characteristics

Table 1 lists the characteristics of the enrolled subjects. There were no age or gender differences between the NSCLC and control groups (P>0.05). In the NSCLC individuals, 193 had AC, 121 had SCC, and 5 had AC and SCC. There were 47 patients in pathological stage I, 55 in stage II, 115 in stage III and 102 in stage IV.

**Table 1:** Clinical characteristics of the subjects enrolled in the present study (Data are mean±SD).

	NSCLC	Control	*P* value
N	319	489	
Ages (years)	55.55±10.69	54.68±10.38	0.25
Sex (M/F)	213/106	313/176	0.42
Adenocarcinoma (AC)	193(60.5%)		
Squamous cell carcinoma (SSC)	121(37.9%)		
Adenocarcinoma and Squamous cell carcinoma (AC+SSC)	5(1.6%)		
Clinical stage			
I	47(14.7%)		
II	55(17.2%)		
III	115(36.1%)		
IV	102(32.0%)		

### 1.2 Association of adiponectin gene promoterSNP-12140G>A, SNP-11426A>G, SNP-11391G>A, and SNP-11377C>G with NSCLC

The allele and genotype frequencies for SNP-12140G>A, SNP-11426A>G, SNP-11391G>A and SNP-11377C>G in the NSCLC and control groups are listed in Table 2. SNP-11391G>A was found to be monomorphic(G allele) in both the NSCLC and control groups. The genotype frequencies for SNP-12140G>A, SNP-11426A>G and SNP-11377C>G were in HWE for the NSCLC and control groups (P>0.05). However, the allele and genotype frequencies of SNP-12140G>A were significantly different in the NSCLC and control groups (P = 0.011 and 0.046, respectively). The G allele for SNP-12140G>A occurred at a significantly higher frequency in the NSCLC group compared with the control group (OR = 1.516; 95% CI: 1.098–2.094). The allele and genotype frequencies for SNP-11426A>G and SNP-11377C>G were not significantly different in the NSCLC and control groups (P>0.05).

**Table 2:** Comparison of genotypic and allelic distribution of SNP-12140G>A, SNP-11426A>G, SNP-11377C>G between NSCLC and control groups.

SNPs	Genotypes[n(%)]			*P* value	Alleles[n(%)]		*P* value	Odds Ratio[%95 CI]
SNP-12140G>A	A/A(freq)	A/G(freq)	G/G(freq)		A(freq)	G(freq)		
NSCLC	4(13.0%)	52(16.3%)	263(82.4%)	0.046	60(9.4%)	578(90.6%)	0.011	1.516[1.098~2.094]
Control	12(2.5%)	109(22.3%)	368(75.3%)		133(13.6%)	845(86.4%)		
**SNP-11426A>G**	**A/A(freq)**	**A/G(freq)**	**G/G(freq)**		**A(freq)**	**G(freq)**		
NSCLC	223(69.9%)	86(27.0%)	10(3.1%)	0.335	532(83.4%)	106(16.6%)	0.169	0.825[0.626~1.086]
Control	360(73.6%)	120(24.5%)	9(1.8%)		840(85.9%)	138(14.1%)		
**SNP-11377C>G**	**C/C(freq)**	**C/G(freq)**	**G/G(freq)**		**C(freq)**	**G(freq)**		
NSCLC	166(52.0%)	129(40.4%)	24(7.5%)	0.853	461(72.3%)	177(27.7%)	0.577	0.938[0.750~1.174]
Control	264(54.0%)	191(39.1%)	34(7.0%)		719(73.5%)	259(26.5%)		

### 1.3 Mode of inheritance analysis of *adiponectin* promoter SNP-12140G>A, SNP-11426A>G and SNP-11377C>G

Tables 3–5 present the results of the mode of inheritance analysis for the four SNPs. To compare each inheritance model (codominant, dominant, recessive, overdominant and log-additive) to the most general model (codominant), the AIC and BIC were calculated to identify the inheritance model that best fit the data[27]. The model with the smallest AIC and BIC value corresponds to the minimal expected entropy[27]. The best fit inheritance model with the lowest AIC and BIC for SNP-12140G>A and SNP-11426A>G was the log-additive model. The best fit inheritance model with the lowest AIC and BIC for SNP-11377C>G was the dominant and log-additive model. The analysis of different genetic models revealed that the GG genotype of SNP-12140G>A conferred more NSCLC risk in the log-additive model. No significant differences for SNP-11426A>G and SNP-11377C>G were found between the NSCLC and control groups in the different genetic models.

**Table 3:** Different inheritance models analysis of the SNP-12140G>A in adiponectin gene promoter between NSCLC and control groups.

Model	Genotype	Control[n(%)]	NSCLC[n(%)]	Before adjusted by Age and Sex		After adjusted by Age and Sex	
				OR (95% CI)	*P* value	OR (95% CI)	*P* value
Codominant	G/G	368 (75.3%)	263 (82.5%)	1.00	0.042	1.00	0.039
	G/A	109 (22.3%)	52 (16.3%)	0.67 (0.46–0.96)		0.67 (0.46–0.96)	
	A/A	12 (2.5%)	4 (1.2%)	0.47 (0.15–1.46)		0.45 (0.14–1.42)	
Dominant	G/G	368 (75.3%)	263 (82.5%)	1.00	0.015	1.00	0.014
	G/A-A/A	121 (24.7%)	56 (17.6%)	0.65 (0.45–0.92)		0.64 (0.45–0.92)	
Recessive	G/G-G/A	477 (97.5%)	315 (98.8%)	1.00	0.220	1.00	0.200
	A/A	12 (2.5%)	4 (1.2%)	0.50 (0.16–1.58)		0.49 (0.16–1.54)	
Overdominant	G/G-A/A	380 (77.7%)	267 (83.7%)	1.00	0.035	1.00	0.035
	G/A	109 (22.3%)	52 (16.3%)	0.68 (0.47–0.98)		0.68 (0.47–0.98)	
Log-additive	—-	—-	—-	0.67 (0.49–0.92)	0.012	0.67 (0.49–0.92)	0.011

**Table 4:** Different inheritance models analysis of the SNP-11426A>G in adiponectin gene promoter between NSCLC and control groups.

Model	Genotype	Control[n(%)]	NSCLC[n(%)]	Before adjusted by Age and Sex		After adjusted by Age and Sex	
				OR (95% CI)	*P* value	OR (95%CI)	*P* value
Codominant	A/A	360 (73.6%)	223 (69.9%)	1.00	0.340	1.00	0.340
	G/A	120 (24.5%)	86 (27%)	1.16 (0.84–1.60)		1.16 (0.84–1.61)	
	G/G	9 (1.8%)	10 (3.1%)	1.79 (0.72–4.48)		1.78 (0.71–4.45)	
Dominant	A/A	360 (73.6%)	223 (69.9%)	1.00	0.250	1.00	0.240
	G/A-G/G	129 (26.4%)	96 (30.1%)	1.20 (0.88–1.64)		1.21 (0.88–1.65)	
Recessive	A/A-G/A	480 (98.2%)	309 (96.9%)	1.00	0.240	1.00	0.250
	G/G	9 (1.8%)	10 (3.1%)	1.73 (0.69–4.30)		1.71 (0.69–4.26)	
Overdominant	A/A-G/G	369 (75.5%)	233 (73%)	1.00	0.440	1.00	0.430
	G/A	120 (24.5%)	86 (27%)	1.13 (0.82–1.57)		1.14 (0.83–1.57)	
Log-additive	—-	—-	—-	1.21 (0.92–1.60)	0.170	1.21 (0.92–1.60)	0.170

**Table 5:** Different inheritance models analysis of the SNP-11377C>G in adiponectin gene promoter between NSCLC and control groups.

Model	Genotype	Control[n(%)]	NSCLC[n(%)]	Before adjusted by Age and Sex		After adjusted by Age and Sex	
				OR (95% CI)	*P* value	OR (95% CI)	*P* value
Codominant	C/C	264 (54%)	166 (52%)	1.00	0.850	1.00	0.840
	G/C	191 (39.1%)	129 (40.4%)	1.07 (0.80–1.44)		1.08 (0.80–1.45)	
	G/G	34 (7%)	24 (7.5%)	1.12 (0.64–1.96)		1.13 (0.65–1.98)	
Dominant	C/C	264 (54%)	166 (52%)	1.00	0.590	1.00	0.570
	G/C-G/G	225 (46%)	153 (48%)	1.08 (0.82–1.43)		1.09 (0.82–1.44)	
Recessive	C/C-G/C	455 (93%)	295 (92.5%)	1.00	0.760	1.00	0.740
	G/G	34 (7%)	24 (7.5%)	1.09 (0.63–1.87)		1.10 (0.64–1.89)	
Overdominant	C/C-G/G	298 (60.9%)	190 (59.6%)	1.00	0.700	1.00	0.680
	G/C	191 (39.1%)	129 (40.4%)	1.06 (0.79–1.41)		1.06 (0.80–1.42)	
Log-additive	—-	—-	—-	1.07 (0.85–1.33)	0.580	1.07 (0.85–1.34)	0.560

### 1.4 Linkage disequilibrium (LD) and haplotype analysis of *adiponectin* promoter SNP-12140G>A, SNP-11426A>G and SNP-11377C>G

Significant LD (D') values between theSNP-12140G>A, SNP-11426A>G and SNP-11377C>G loci were found in all individuals. The D' value was 0.899 between SNP-12140G>A and SNP-11426A>G, 0.857 between SNP-12140G>A and SNP-11377C>G and 0.864 between SNP-11426A>G and SNP-11377C>G. Based on the LD result, we calculated the haplotypes of these three SNPs, and Table 6 shows the estimated frequencies of SNP-12140G>A-SNP-11426A>G-SNP-11377C>G haplotypes with frequencies of more than 3%. None of the haplotypes were significantly different in the NSCLC and control groups after Bonferroni correction (P>0.05).

**Table 6:** Different haplotypes analysis of SNP-12140G>A, SNP-11426A>G, SNP-11377C>G in adiponectin gene promoter between NSCLC and control groups.

SNP-12140G>A	SNP-11426A>G	SNP-11377C>G	NSCLC[n(%)]	Control[n(%)]	*P* value	*P* value	OR	[%95 CI]
					Fisher's	Bonferroni correction		
A	A	C	59.97(9.4%)	127.20(13.0%)	0.025	>0.05	0.691	[0.499~0.956]
A	A	G	0.03(0.0%)	5.45(0.6%)	-	-	-	-
A	G	C	0.00(0.0%)	0.35(0.0%)	-	-	-	-
G	A	C	298.53(46.8%)	456.69(46.7%)	0.980	>0.05	0.997	[0.816~1.219]
G	A	G	173.47(27.2%)	250.67(25.6%)	0.511	>0.05	1.079	[0.860~1.353]
G	G	C	102.50(16.1%)	134.76(13.8%)	0.214	>0.05	1.193	[0.903~1.577]
G	G	G	3.50(0.5%)	2.89(0.3%)	-	-	-	-

### 1.5 Association analysis of *adiponectin* promoter SNP-12140G>A, SNP-11426A>G, and SNP-11377C>G with different pathologic stages

There were no differences in the allele and genotype frequencies forSNP-12140G>A, SNP-11426A>G and SNP-11377C>G between pathologic stages I+II and III+IV (Table 7).

**Table 7:** Comparison of genotypic and allelic distribution of SNP-12140G>A, SNP-11426A>G, SNP-11377C>G between pathologic stages I+II and III+IV.

SNPs	Genotypes[n(%)]			*P* value	Alleles[n(%)]		*P* value	Odds Ratio[%95 CI]
**SNP-12140G>A**	**A/A(freq)**	**A/G(freq)**	**G/G(freq)**		**A(freq)**	**G(freq)**		
Stage I+II	3(2.9%)	18(17.6%)	81(79.4%)	0.154	24(11.8%)	180(88.2%)	0.161	0.678[0.393~1.171]
Stage III+IV	1(0.5%)	34(15.7%)	182(83.9%)		36(8.3%)	398(91.7%)		
**SNP-11426A>G**	**A/A(freq)**	**A/G(freq)**	**G/G(freq)**		**A(freq)**	**G(freq)**		
Stage I+II	70(68.6%)	27(26.5%)	5(4.9%)	0.463	167(81.9%)	37(18.1%)	0.479	1.172[0.755~1.819]
Stage III+IV	153(70.5%)	59(27.2%)	5(2.3%)		365(84.1%)	69(15.9%)		
**SNP-11377C>G**	**C/C(freq)**	**C/G(freq)**	**G/G(freq)**		**C(freq)**	**G(freq)**		
Stage I+II	50(49.0%)	45(44.1%)	7(6.9%)	0.653	145(71.1%)	59(28.9%)	0.649	1.090[0.753~1.576]
Stage III+IV	116(53.5%)	84(38.7%)	17(7.8%)		316(72.8%)	118(27.2%)		

### 1.6 Association analysis of *adiponectin* promoter SNP-12140G>A, SNP-11426A>G, and SNP-11377C>G with adenocarcinoma (AC) and squamous cell carcinoma (SCC)

Our results revealed no differences in the allele and genotype frequencies for SNP-12140G>A, SNP-11426A>G and SNP-11377C>G between AC and SCC(Table 8).

**Table 8:** Association analysis of the adiponectin promoter SNP-12140G>A, SNP-11426A>G, SNP-11377C>G between adenocarcinoma (AC) and squamous cell carcinoma (SCC).

SNPs	Genotypes[n(%)]			*P* value	Alleles[n(%)]		*P* value	Odds Ratio[%95 CI]
**SNP-12140 G>A**	**A/A(freq)**	**A/G(freq)**	**G/G(freq)**		**A(freq)**	**G(freq)**		
AC[n(%)]	3(1.6%)	31(16.1%)	159(82.4%)	0.850	37(9.6%)	349(90.4%)	0.702	1.116[0.636~1.956]
SCC[n(%)]	1(0.8%)	19(15.7%)	101(83.5%)		21(8.7%)	221(91.3%)		
**SNP-11426 A>G**	**A/A(freq)**	**A/G(freq)**	**G/G(freq)**		**A(freq)**	**G(freq)**		
AC[n(%)]	138(71.5%)	49(25.4%)	6(3.1%)	0.591	325(84.2%)	61(15.8%)	0.363	1.217[0.797~1.860]
SCC[n(%)]	80(66.1%)	37(30.6%)	4(3.3%)		197(81.4%)	45(18.6%)		
**SNP-11377 C>G**	**C/C(freq)**	**C/G(freq)**	**G/G(freq)**		**C(freq)**	**G(freq)**		
AC[n(%)]	101(52.3%)	80(41.5%)	12(6.2%)	0.617	282(73.1%)	104(26.9%)	0.668	1.081[0.756~1.548]
SCC[n(%)]	63(52.1%)	47(38.8%)	11(9.1%)		173(71.5%)	69(28.5%)		

## Discussion

Recent evidence has demonstrated that adiponectin has antiproliferative and proapoptotic effects in breast cancer cell lines in vitro[9, 10]. In 2007, Petridou *et al*. found that serum adiponectin was not significantly different in patients with lung cancer compared with controls but that it was significantly lower in patients at advanced disease stages, suggesting that adiponectin could be a potential marker for lung cancer progression[3].Pei *et al*. then undertook a meta-analysis to exploit the causal relevance of circulating adiponectin with cancer; their findings demonstrated that genetically higher circulating adiponectin conferred a protective effect against lung cancer but a risk for colorectal cancer[29].

In 2011, Cui *et al*. investigated the association of SNPs in the adiponectin gene (rs266729, rs822395, rs822396 and rs2241766) with NSCLC[30], and the SNPs they chose were reported to be related to circulating adiponectin levels[30]. Although Cui *et al*. failed to observe an association for SNP-11377C>G (rs266729), which is located in the *adiponectin* promoter, with NSCLC[30], their results revealed that the rs2241766 TT genotype was significantly associated with NSCLC susceptibility. In 2006, Heid *et al*. reported that rs2241766 was associated with adiponectin levels, with the GG genotype correlating with increased adiponectin levels compared with the TT genotype [21]. In addition, Pei *et al*. also observed that the rs2241766 GG genotype or G allele was associated with significantly higher circulating adiponectin levels than the TT genotype, without heterogeneity[29]. Moreover, a meta-analysis of cancer risk and adiponectin gene polymorphisms by Xu *et al*. and Yang *et al*. indicated that the G allele of rs2241766 was a potential protection factor for cancer risk[31, 32]. Therefore, rs2241766TTisassociated with lower adiponectin levels, and thers2241766TT genotype may be significantly associated with NSCLC susceptibility.

In 2009, Laumen *et al*. found that SNP-11426A>G, SNP-11391G>Aand SNP-11377C>G within the *adiponectin* promoter were regulatory SNPs for *adiponectin* promoter activity[22]. Consequently, we choose four *adiponectin* promoter SNPs to evaluate the association of these SNPs and their haplotypes with NSCLC in a Han Chinese population. Our results confirmed our previous finding that the G allele of the *adiponectin* promoter SNP-12140G>A is a risk factor for NSCLC[23] and that individuals with the SNP-12140G>A GG genotype have increased risk for NSCLC under log-addictive models. The other three adiponectin gene promoter SNPs, including SNP-11377C>G, were not correlated with NSCLC in the current study. In 2008, Kaklamani *et al*. reported that adiponectin gene promoter SNP-11377C>G is associated with colorectal cancer risk[33]. However, Carvajal-Carmona *et al*. failed to observe an association between SNP-11377C>G and colorectal cancer risk in patients from the UK[34], stating that the discrepancy between their results andthose of Kaklamani *et al*. may be due to differences between ethnic groups, differential environmental effects or false-positive results[34]. Pei *et al*. reported an interesting finding that genetically elevated circulating adiponectin may confer a protective effect against lung cancer but a risk for colorectal cancer[29]. Thus, the type of cancer could be a key factor in the relationship between cancer and the adiponectin gene. Our results, together with those of Cui *et al*. [30], indicated that SNP-11377C>G is not associated with NSCLC risk and suggested that the role of adiponectin gene SNP-11377C>G may be specific to the cancer type or ethnicity. Additionally, our results indicated that the role of adiponectin gene promoter SNPs may be specific to the SNP site, e.g.,SNP-12140G>A maybe more important than SNP-11377C>G in the Chinese population. Based on these results, we hypothesized that SNP-12140G>A may influence *adiponectin* promoter activity, gene expression, plasma adiponectin level and, ultimately, NSCLC risk. Unfortunately, to date, the role of SNP-12140G>A in *adiponectin* promoter activity and gene expression has not been tested. Therefore, the function of this SNP in *adiponectin* promoter activity and gene expression should be investigated in the future. It was an interesting finding that only SNP-12140G>A was associated with NSCLC in our study. Our data showed that SNP-12140G>A just was partly in linkage disequilibrium with SNP-11426A>G and SNP-11377C>G. Therefore, we assumed this could be possible one of causes of the divergence that only SNP-12140G>A associated with NSCLC in this Han Chinese population, but not SNP-11426A>G and SNP-11377C>G.

In this study, we also observed that there were no differences in allele and genotype frequency for SNP-12140G>A, SNP-11426A>G and SNP-11377C>G between AC and SCC (Table 8). In addition, there were no differences in allele and genotype frequency for SNP-12140G>A, SNP-11426A>G and SNP-11377C>G between pathologic stages I+II and III+IV (Table 7). However, in 2007, Petridou *et al*. found that adiponectin level was not significantly different between lung cancer individuals compared with controls, though it was significantly lower in patients with advanced disease stage[3]. These author spostulated that the reason that a significantly lower adiponectin level was observed in advanced disease-stage patients may be due to the decrease in overall fat mass in advanced lung cancer, leading to the decreased production of adiponectin by subcutaneous adipose tissue [3].Thus, SNP-12140G>A may be merely a susceptibility factor for NSCLC in the Han Chinese population and not associated with lung cancer stage. The association of lower adiponectin with advanced disease stage could be the due to the decreased production of adiponectin induced by the decrease in fat mass in advanced lung cancer stages. Because we did not measure adiponectin levels in the NSCLC and control groups, which is a limitation of our study, we did not observe an association between genetic data, adiponectin levels and NSCLC risk and NSCLC pathologic stage.

There were other several limitations in the present study. A relatively small sample size may limit the statistical power of our study. The statistical power for the effect of SNP-12140G>A was calculated using PS software [28],and we found that our sample reached 74.1% of statistical efficacy. Thus, a larger population should be investigated to further verify our results. The other limitation of this study was that we did not ascertain the smoking status of the control individuals, making it difficult to perform future analyses of such exposure variables and to perform a gene-smoking interaction analysis. This limitation may neutralize the effect of smoking and expose the effects of genetic variants in our study.

## Conclusions

In this study, we performed an association study for *adiponectin* promoter SNP-12140G>A, SNP-11426A>G and SNP-11377C>G and NSCLC in a Han Chinese population and found that the G allele of SNP-12140G>A is a risk factor for NSCLC; individuals with the SNP-12140G>A GG genotype showed increased risk for NSCLC under log-addictive models. In the future, larger scale studies are needed to better clarify and examine the association of adiponectin gene promoter variations with NSCLC susceptibility. Moreover, the function of SNP-12140 in *adiponectin* promoter activity and gene expression should be investigated.

## Supporting Information

S1 Table (XLSX)

S2 Table (XLSX)
